# Zoledronic Acid Implant Coating Results in Local Medullary Bone Growth

**DOI:** 10.1021/acs.molpharmaceut.2c00644

**Published:** 2022-11-15

**Authors:** Juliana C. Quarterman, Pornpoj Phruttiwanichakun, Douglas C. Fredericks, Aliasger K. Salem

**Affiliations:** † Department of Pharmaceutical Sciences and Experimental Therapeutics, College of Pharmacy, University of Iowa, Iowa City, Iowa 52242, United States; ‡ The Bone Healing Research Laboratory, Department of Orthopedics and Rehabilitation, Carver College of Medicine, University of Iowa, Iowa City, Iowa 52242, United States

**Keywords:** zoledronate, PLGA, titanium implant, bone regeneration, osteoarthritis

## Abstract

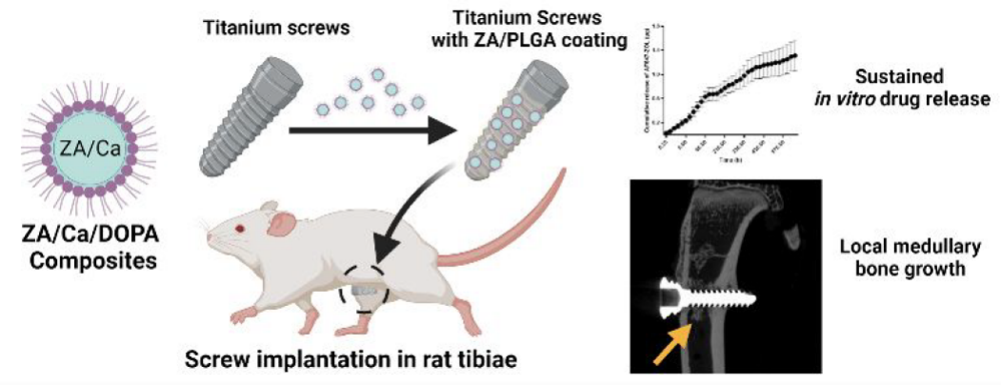

Osteoarthritis (OA) can necessitate surgical interventions to restore the function of the joint in severe cases. Joint replacement surgery is one of the procedures implemented to replace the damaged joint with prosthetic implants in severe cases of OA. However, after successful implantation, a fraction of OA patients still require revision surgery due to aseptic prosthetic loosening. Insufficient osseointegration is one of the factors that contribute to such loosening of the bone implant, which is commonly made from titanium-based materials. Zoledronic acid (ZA), a potent bisphosphonate agent, has been previously shown to enhance osseointegration of titanium implants. Herein, we fabricated ZA/Ca composites using a reverse microemulsion method and coated them with 1,2-dioleoyl-*sn*-glycero-3-phosphate monosodium salt (DOPA) to form ZA/Ca/DOPA composites. Titanium alloy screws were subsequently dip-coated with a suspension of the ZA/Ca/DOPA composites and poly(lactic-*co*-glycolic) acid (PLGA) in chloroform to yield Za/PLGA-coated screws. The coated screws exhibited a biphasic *in vitro* release profile with an initial burst release within 48 h, followed by a sustained release over 1 month. To assess their performance *in vivo*, the Za/PLGA screws were then implanted into the tibiae of Sprague–Dawley rats. After 8 weeks, microCT imaging showed new bone growth along the medullary cavity around the implant site, supporting the local release of ZA to enhance bone growth around the implant. Histological staining further confirmed the presence of new mineralized medullary bone growth resembling the cortical bone. Such local medullary growth represents an opportunity for future studies with alternative coating methods to fine-tune the local release of ZA from the coating and enhance complete osseointegration of the implant.

## 1 Introduction

Osteoarthritis (OA) is a degenerative and oftentimes age-dependent bone disease which affects millions worldwide.^1,2^ This disease affects all aspects of the joint including but not limited to the articular cartilage, subchondral bone, and associated ligaments and causes joint failure.^3^ These deleterious changes to the cartilage and subchondral bone are the main causes for pain and loss of mobility in patients.^3^ OA management options are mostly relegated to pain management using either pharmacological means such as oral and topical nonsteroidal anti-inflammatory drugs and viscosupplementation injections of hyaluronic acid or nonpharmacological means such as exercise and weight loss.^3,4^ However, in the case of patients with end stage OA who do not have improved outcomes after trying these management options, surgical intervention, or arthroplasty, is often the next step.^3−5^ Though it is possible for OA to present in the knee, elbow, and ankle joints, the most common sites where OA reaches the point for arthroplasty are in the hip and knee.^5−7^ Joint replacement surgery is a procedure where the surgeon will remove the damaged bone and cartilage and use a prosthesis to restore normal functionality and alignment to the joint.^5^ To ensure the bone implant adheres strongly and quickly to the bone such that there is minimal movement of the implant, several factors must be considered including that the materials chosen for the implant are (1) biocompatible, (2) able to integrate with the host bone tissue, (3) nonimmunogenic, and (4) able to provide mechanical and structural support which indirectly promotes bone healing due to the reduced shear stress.^8,9^ Bone implants have been engineered using bulk materials made from biomaterials such as hydroxyapatite,^10^ degradable polymers such as poly(lactic-*co*-glycolic) acid (PLGA) or polycaprolactone;^11,12^ or made from metallic materials, such as magnesium,^13^ stainless steel,^14^ titanium,^15^ or any combinations of these materials.^16^ Titanium and its alloys are the most commonly used materials for bone implants as they possess high mechanical strength, are anticorrosive, have a high elastic modulus and high biocompatibility, and are permissive to bone apposition owing to their bioinertness.^17,18^ In addition, titanium-based implants have been shown to cause decreased fibrous tissue formation around the implant compared to other metals.^17^ Though joint replacement surgeries have a high rate of success, there is a need for revision surgery within the first 25 years after implantation in 20% of cases.^19,20^ The most common cause for this implant failure is aseptic prosthetic loosening, and there have been several strategies aimed at avoiding this issue.^4,21,22^ Implant failure can be the result of several contributing factors such as inadequate qualitative or quantitative bone stock, as well as age, gender, implant insertion trauma, and/or poor osseointegration.^9,18^ For this work, we were focused on poor osseointegration as a contributing factor to aseptic prosthetic loosening.^23^

Osseointegration is the process by which the implant surface forms a direct bond with the surrounding bone tissue.^24,25^ Depending on the implant site, a bone implant will need to meet specific osseointegration requirements.^8^ For example, fixation screws and plates that are removed from the body following healing have low to moderate osseointegration requirements.^8^ Other implants such as pedicle screws used in spinal fusion surgeries or joint replacements need to remain for the lifetime of the patient to provide support and hence have high osseointegration requirements.^8,26^ Several approaches have been investigated to improve the osseointegration of implants, including the use of bioactive bulk materials^27,28^ or making physicochemical modifications to the implant surface.^8,28,29^ However, in this work we were focused on the use of bisphosphonates for their antiresorptive properties.^8,30,31^ Bisphosphonates are potent inhibitors of osteoclast-mediated bone resorption and are most commonly used to treat bone disorders such as osteoporosis, Paget’s disease, and osteogenesis imperfecta.^31^ Bisphosphonates are uniquely qualified for bone disease treatment because of their high affinity for bone tissue compared to other tissues.^31,32^ Since one of the distinctive aspects of post-traumatic osteoarthritis (PTOA) progression is remodeling of the subchondral bone, bisphosphonate compounds are a promising candidate for OA treatment due to their antiresorptive properties.^30^ Bisphosphonates can be broadly divided into two groups, namely, nitrogen-containing and non-nitrogen-containing, where the nitrogen-containing bisphosphonates are the more potent of the two due to their higher affinity for calcium hydroxyapatite.^31^ Zoledronic acid (ZA) is a nitrogen-containing bisphosphonate compound and was chosen for our studies because it is already used in clinical practice, being delivered via infusion, and has been shown to promote osseointegration of titanium implants in ovariectomized rabbits.^33^ It has been shown that bisphosphonates can induce osteoclast apoptosis, decrease osteoclastogenesis, and increase the function of osteoblasts.^31^ In prior research, bisphosphonates, such as ZA, have been delivered via intravenous,^34,35^ subcutaneous,^33^ intraperitoneal,^36^ or topical routes.^23^ Local release of bisphosphonate(s) has been investigated by several research groups to enhance fixation and osseointegration of bone implants.^37−45^ It has been shown that, without additional coating agents or surface modification, bisphosphonate-coated titanium screws exhibited a fast *in vitro* bisphosphonate release profile with most of the drug being released within the first 3 days, signifying the importance of implant coating in the control of bisphosphonate release.^45^ In contrast, sustained local delivery of bisphosphonate(s) has been achieved by incorporating bisphosphonate(s) into the coating layers of implants via various techniques, such as hydroxyapatite (HA),^37,38^ immobilized fibrinogen,^38−40^ and poly(d,l-lactic acid) (PDLLA) coatings.^41−44^ Methods such as the ZA/PDLLA coating technique still suffer from a burst release *in vitro*, with ∼90% of the loaded ZA being released within the first 24 h,^41^ prompting a need for the development of a new coating technique that provides sustained release of ZA.

To date, ZA has not been incorporated into a PLGA for the purposes of bone implant coating. PLGA is an FDA-approved biodegradable polymer that has been used in several commercially available drug delivery systems.^11,30,46−50^ PLGA is uniquely qualified as a drug delivery vehicle because its release kinetics can be finely tuned to fit specific needs by varying the ratio of lactic acid to glycolic acid monomers.^51^ The decision to deliver drugs locally has several advantages over systemic administration such as (1) decreased risk of systemic side effects, (2) the ability to deliver multiple therapeutics at a time, and (3) fewer and often lower doses being necessary.^9^ We hypothesized that delivering ZA locally using a biodegradable polymer matrix bone implant coating would result in improved implant osseointegration and decreased risk of adverse side effects due to the lowered drug concentration needed in the formulation compared to systemic administration.

In our studies, bisphosphonate-metal composites comprising CaCl_2_, ZA, and/or the fluorescently labeled version of ZA (AF647-ZOL) were synthesized. The composites were then coated in the lipid 1,2-dioleoyl-*sn*-glycero-3-phosphate monosodium salt (DOPA). The composites were characterized for drug content, hydrodynamic diameter, and zeta potential. The various composites were then used to dip-coat model titanium alloy screws which were then assayed for drug deposition, *in vitro* release profiles, and finally used in an *in vivo* rat study to evaluate the effectiveness of the ZA/PLGA-coated screws compared to uncoated or PLGA only coated screws to induce bone implant osseointegration.

## 2 Materials and Methods

### 2.1 Materials

Zoledronic acid (1-hydroxy-2-(1-imidazolyl) ethane-1,1-diphosphonic acid monohydrate) was purchased from Tokyo Chemical Industry Co., Ltd. America (Portland, OR). Nanopure water was obtained from a Barnstead Nanopure Diamond purification system (ThermoFisher Scientific, Waltham, MA). Ammonium hydroxide was purchased from ThermoFisher Scientific. Formic acid and calcium chloride (CaCl_2_) were purchased from J. T. Baker Chemical (Austin, TX). Cyclohexane was purchased from ThermoFisher Scientific. Polyoxyethylene(5)nonylphenylether (NP-5, IGEPAL CO-520) was purchased from Sigma-Aldrich (St. Louis, MO). The lipids 1,2-dioleoyl-*sn*-glycero-3-phosphate monosodium salt (DOPA) and 1,2-distearoyl-*sn*-glycero-3-phosphoethanolamine-*N*-[methoxy (polyethylene glycol)-2000 (DSPE-PEG_2K_) were purchased from Avanti Polar Lipids (Alabaster, AL). Ethanol and chloroform were purchased from ThermoFisher Scientific. Poly(lactic-*co*-glycolic) acid (PLGA, Resomer RG 503) was purchased from Evonik (Darmstadt, DEU). AF647-ZOL was purchased from BioVinc (Pasadena, CA). Titanium screw implants (Ti-6AL-4 V S-T cortex screw 1.5 mm × 10 mm) used for *in vitro* studies were purchased from Smith & Nephew (Memphis, TN). Locking screws (1.5 × 8 mm^2^) used for the *in vivo* rat studies were purchased from TDM USA (Salt Lake City, Utah). EmbryoMax 1× Dulbecco’s phosphate-buffered saline (PBS) was purchased from Sigma-Aldrich (St. Louis, MO).

### 2.2 Instrumentation

Ultraviolet–visible (UV–vis) spectrophotometer measurements were performed using a SpectraMax M5 multimode plate reader (Molecular Devices, San Jose, CA). HPLC measurements were carried out using a 2690 Alliance HPLC separation module coupled with a 2487 dual λ absorbance UV–vis detector (Waters, Milford, MA). Analytes were weighed using a Mettler Toledo XS104 analytical balance (Mettler-Toledo, Columbus, OH). Fluorescence spectroscopy measurements were performed using a SpectraMax M5 multimode plate reader (Molecular Devices, San Jose, CA).

### 2.3 Analytical and Chromatographic Conditions

Determination of ZA concentration in samples was performed using IE-HPLC. A Dionex IonPac AS7 analytical column (10 μm, 4 × 250 mm^2^, ThermoFisher Scientific) equipped with a guard column (10 μm, 4 × 250 mm^2^, ThermoFisher Scientific) was utilized. The mobile phase was pumped at an isocratic flow rate of 1.6 mL/min (min). The detection wavelength was 215 nm, the injection volume was 80 μL, and the run time was 10 min. Chromatographic separation of ZA was performed at room temperature (RT) for all injected samples.

### 2.4 Mobile Phase Preparation

The mobile phase consisted of 0.2% formic acid brought up to pH 3.0 using aqueous ammonium hydroxide solution (∼6.2 mL, 1.48 N). The mobile phase was degassed using an ultrasonic bath (Branson Ultrasonics, Danbury, CT). The pH was measured using a three-point calibrated pH meter (Mettler-Toledo SevenEasy S20; Columbus, OH).

### 2.5 Sample Preparation

#### 2.5.1 Stock Solutions, Working Solutions, Calibration Standards, and Quality Control Samples

ZA stock solutions were prepared in the mobile phase (0.2% formic acid, pH 3.0) to give a final concentration of 500 μg/mL, and all subsequent dilutions were made using the mobile phase.

AF647-ZOL samples were measured for concentration by fluorescence spectroscopy using excitation and emission wavelengths of 648 and 666 nm, respectively. The concentration of samples was determined by comparing the absorbance of the samples to that obtained from a standard curve of known concentrations.

### 2.6 Synthesis of ZA/Ca/DOPA, ZA/AF647-ZOL/Ca/DOPA, and AF-ZOL/Ca/DOPA Composites

**Figure 1:**
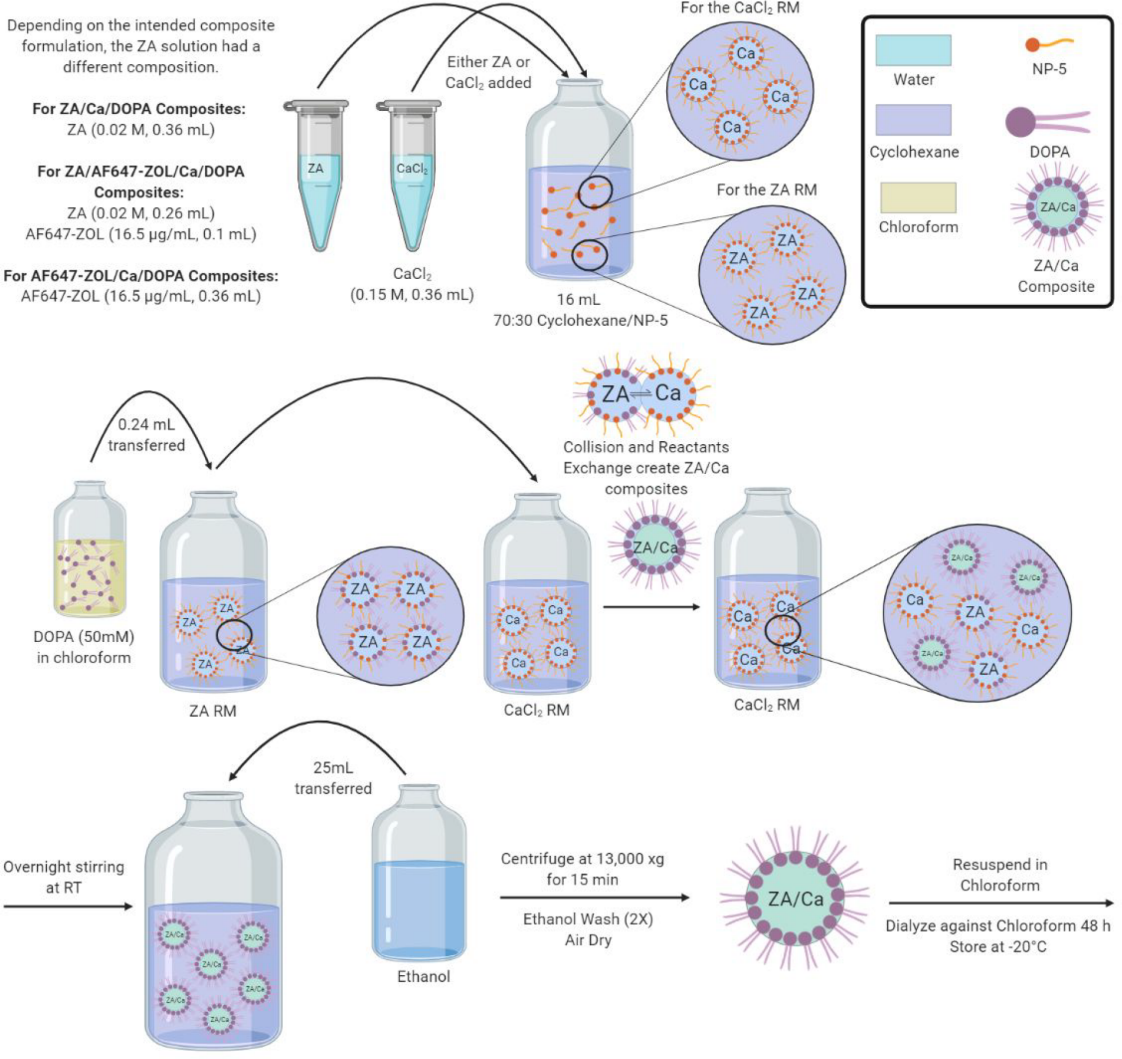
Schematic representation of the synthesis process for ZA/Ca/DOPA, AF647-ZOL/Ca/DOPA, and ZA/AF647-ZOL/Ca/DOPA composites. Further description can be found in section 2.6.

ZA/Ca/DOPA composites were synthesized (Figure 1) as previously described in the literature^52−55^ and were used to coat screws for the *in vivo* animal study. Briefly, aqueous solutions of ZA and CaCl_2_ were made at concentrations of 0.02 and 0.15 M, respectively. Next, 360 μL of either solution was added dropwise to 16 mL of cyclohexane/NP-5 (70:30) with magnetic stirring, followed by sonication for several minutes until the reverse microemulsion (RM) was transparent. A solution of DOPA in chloroform was made to a concentration of 50 mM, and 240 μL was added dropwise with magnetic stirring to the ZA RM. This mixture was sonicated for several minutes, left stirring for 10 min, then immediately added to the CaCl_2_ RM. The reaction was left stirring overnight, and after at least 16 h, 25 mL of ethanol was added to the RM to cause the precipitation of the ZA/Ca/DOPA composites. The precipitate was collected using centrifugation at 13,000*g* for 15 min (Sorvall Legend XTR Centrifuge; ThermoFisher Scientific), washed twice with ethanol, and air-dried. The precipitate was then resuspended in 2 mL of chloroform, syringe filtered using a Choice PTFE (hydrophobic) syringe filter (ThermoFisher Scientific) and dialyzed against chloroform for 48 h using benzoylated dialysis tubing (2 kDa molecular weight (MW) cutoff; Sigma-Aldrich). The resulting suspension was stored at −20 °C until use.

The ZA/AF647-ZOL/Ca/DOPA composites were synthesized using a modified method to that in the literature.^52−55^ These composites were used to coat screws for determining the drug deposition, and the ZA RM consisted of 260 μL of the ZA in water at a concentration of 0.02 M combined with 100 μL of AF647-ZOL in water dissolved at a concentration of 16.484 μg/mL. The rest of the synthesis procedure was the same as that for ZA/Ca/DOPA composites. AF647-ZOL, due to its similar *in vivo* biodistribution to ZA, has been used in multiple studies to enable *in vivo* biodistribution studies of the bisphosphonate in its free form and in nanoparticulate delivery systems.^52,53,56−58^ AF647-ZOL has also been reported to provide very similar *in vitro* release kinetics to ZA when prepared as bisphosphonate–metal composites,^52^ hence allowing for the use of AF647-ZOL in our *in vitro* release study and IVIS imaging of the coated screws for sensitive detection and quantification due to its fluorescent nature.

The AF647-ZOL/Ca/DOPA composites that were used to coat the screws for the *in vitro* release study were synthesized using a modified method to that in the literature.^52−55^ For these composites, 360 uL of AF647-ZOL in water at a concentration of 16.484 μg/mL was used to create the ZA RM. The rest of the synthesis procedure was the same as that for ZA/Ca/DOPA composites.

**Figure 2:**
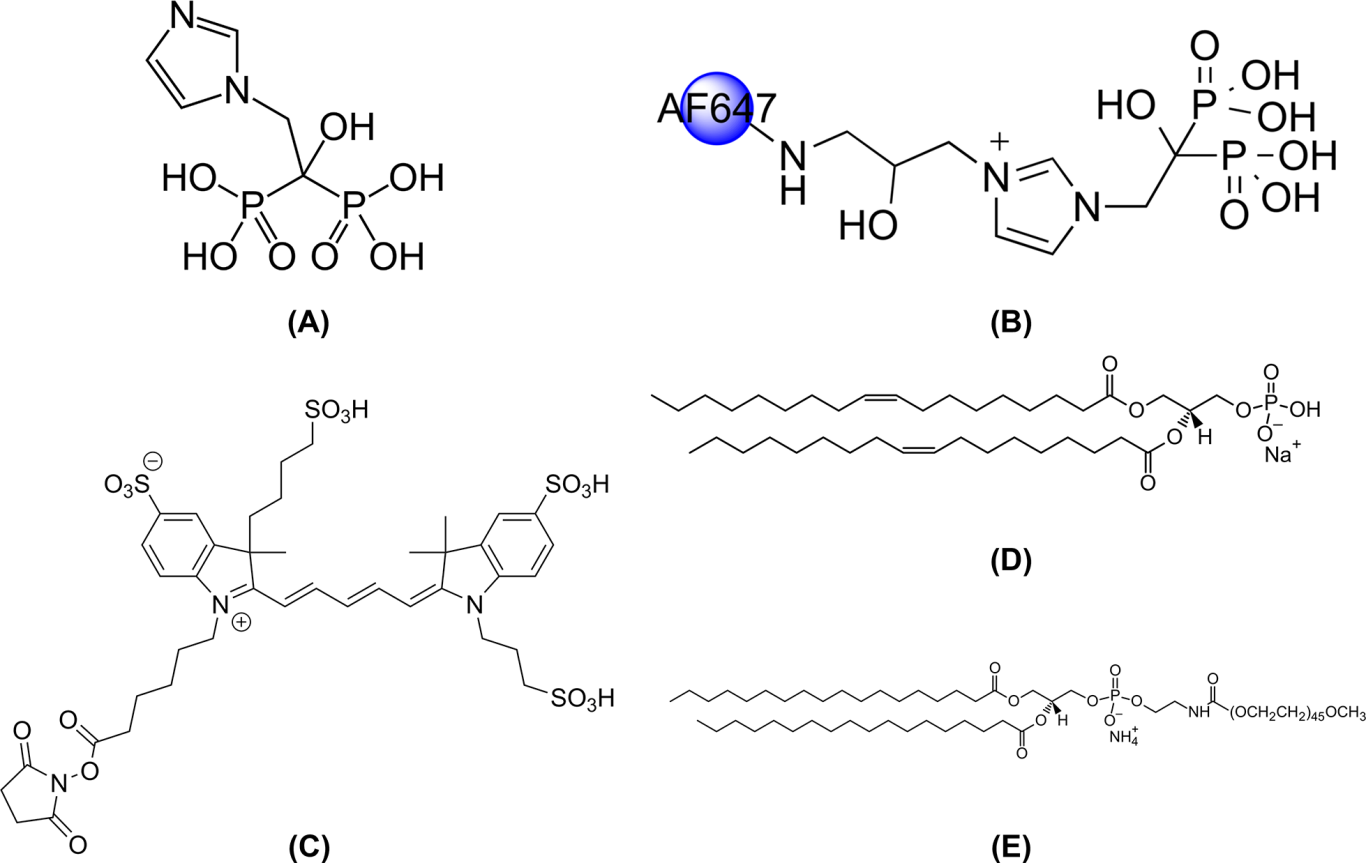
Chemical structures for (A) ZA; (B) AF647-ZOL; (C) AlexaFluor647 fluorescence probe that is chemically bound to the ZA molecule to create the AF647-ZOL compound; (D) DOPA (1,2-dioleoyl-*sn*-glycero-3-phosphate (sodium salt)); and (E) DSPE-PEG_2K_ (1,2-distearoyl-*sn*-glycero-3-phosphoethanolamine-*N*-[methoxy(polyethylene glycol)-2000] (ammonium salt)).

The ZA/AF647-ZOL/Ca/DOPA and AF647-ZOL/Ca/DOPA composites were prepared in darkened rooms to avoid light exposure. Once the synthesis was completed, composite samples were stored in aluminum foil covered glass screw top vials, and any additional handling of the composites was done in darkened rooms and aluminum foil covered glassware. Figure 2A–D shows the chemical structures for ZA, AF647-ZOL, the attached AlexaFluor647 fluorescent probe, and DOPA, respectively.

### 2.7 Characterization of ZA/Ca/DOPA, ZA/AF647-ZOL/Ca/DOPA, and AF-ZOL/Ca/DOPA Composites

The concentrations of ZA in both the ZA/Ca/DOPA and ZA/AF647-ZOL/Ca/DOPA composites were determined using the previously described IE-HPLC method (section 2.3). Samples were prepared for injection by first rendering composites water-soluble by combining 100 μL of the composite suspension with 1 mg of the net neutral lipid DSPE-PEG_2K_ (Figure 2E). The chloroform was removed using rotary evaporation (40 mbar, 30 min, Rotavapor R-300; Büchi, Flawil, CHE).

The composites were resuspended in 0.5 mL of the mobile phase (0.2% formic acid, pH 3.0) for drug content analysis or resuspended in 1 mL of the mobile phase for hydrodynamic size, polydispersity index (PDI), and zeta potential measurements. The ZA concentration was determined by comparing the AUC of the sample to a calibration curve of known ZA concentrations dissolved in the mobile phase (0.2% formic acid, pH 3.0). The percent encapsulation efficiency (%EE) was found using the following formula.
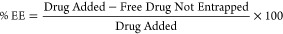


In order to characterize the morphology of the ZA/Ca/DOPA composites, a transmission electron microscopy (TEM) (JEM 1230 JEOL USA; Peabody, MA) operated at 120 kV located at the Central Microscopy Research Facility at the University of Iowa was used.

The AF647-ZOL concentration in the ZA/AF647-ZOL/Ca/DOPA and AF647-ZOL/Ca/DOPA composites was determined by comparing a standard curve of known AF647-ZOL concentrations to a sample of the DSPE-PEG_2K_-coated composites using fluorescence spectroscopy. The data was plotted using GraphPad Prism version 9.0.0.

A Zetasizer Nano ZS particle analyzer (Malvern Panalytical Ltd., Westborough, MA) was used to measure the hydrodynamic diameter, PDI, and zeta potential for the ZA/Ca/DOPA, ZA/AF647-ZOL/Ca/DOPA, and AF647-ZOL/Ca/DOPA composites after coating in DSPE-PEG_2K_ and resuspension in 1 mL of Nanopure water. This data was plotted using GraphPad Prism version 9.0.0.

### 2.8 Coating Composition and Technique

The ZA/AF67-ZOL/PLGA, AF67-ZOL/PLGA, and ZA/PLGA coating solutions were prepared in 2 mL glass vials (8 mm screw thread; ThermoFisher Scientific) by combining 400 μL of a 30% (*w*/*v*) PLGA in chloroform with 200 μL of the requisite composite suspension depending on the purpose of the coating solution. The coating was applied to the model titanium screws using a dip-coating technique where the screw was lowered into the solution for 10 s and removed from the solution over 10 s. Each coat was air-dried for 10 min between coatings and the screws were coated seven times total. The screws were air-dried at RT overnight before use in any subsequent experiment.

In order to make the PLGA only coating solution, 400 μL of a 30% (*w*/*v*) PLGA in chloroform was combined with 200 μL of chloroform. The coating method was the same as was performed for the ZA/AF67-ZOL/PLGA, AF67-ZOL/PLGA, and ZA/PLGA coating solutions.

It is important to note that in order to have a higher drug concentration for the *in vivo* study ZA/PLGA coating solution, 11 batches of ZA/Ca/DOPA composites suspensions in chloroform were combined, the chloroform removed using rotary evaporation, and then the dried precipitate was resuspended in 2 mL of chloroform. This ZA/Ca/DOPA composite stock solution was then used to create the ZA/PLGA coating solution.

### 2.9 IVIS Imaging of ZA/AF647-ZOL/PLGA-Coated Screws

The amount of drug deposited onto a ZA/AF647-ZOL/PLGA-coated screw was determined by measuring the fluorescence of the screw using an IVIS Lumina S5 instrument (PerkinElmer, Waltham, MA) equipped with Living Image software (Small Animal Imaging Core Facility; University of Iowa). The fluorescence intensities of the ZA/AF647-ZOL/PLGA-coated screws, uncoated screw, and remaining coating solution were determined after the dip-coating process was completed. Since the screw was laid on its side when being imaged, the value for the fluorescence intensity was doubled to account for the fact that only half of the screw was visible to the camera. These intensity values were compared to that obtained from imaging AF647-ZOL in water solutions with known concentrations ranging from 0.211 ng/mL to 0.132 μg/mL along with a blank containing only Nanopure water. These samples were used as references against which the screw and coating solution samples could be compared in order to determine the amount of drug deposited onto the screw surface. The absorbance wavelength used was 620 nm, and the emission wavelength measured was 670 nm. The data was plotted using GraphPad Prism version 9.0.0.

### 2.10 Release Study Using AF647-ZOL/PLGA-Coated Screws

In order to determine the release profile for AF647-ZOL/PLGA-coated screws, six screws were dip-coated using the above-mentioned coating technique. After overnight air drying, each screw was placed into a 24-well plate (1 screw/well) and 600 μL of PBS was added to each well with the plate left stirring at room temperature. At each predetermined time point, the entirety of the release media was removed from the well and replaced with fresh media. The amount of drug released at each time point was measured using fluorescence spectroscopy (SpectraMax M5 multimode plate reader; Molecular Devices, San Jose, CA) by adding 100 μL of each sample to a black clear optical bottom 96 well plate (ThermoFisher Scientific). The fluorescence intensities of these samples were used to determine the sample’s drug content by comparing it to a standard curve of known AF647-ZOL concentrations ranging from 0.016 to 0.258 μg/mL. The absorbance wavelength used was 648 nm, and the emission wavelength measured was 666 nm. The data from the release study are reported as the cumulative amount of AF647-ZOL released from all six coated screws versus time and were plotted using GraphPad Prism version 9.0.0.

### 2.11 Animal Study Protocol

The comparative effectiveness of the ZA/PLGA coating versus PLGA only and uncoated screws was evaluated using a rat model. All animal experiments were done in accordance with the University of Iowa Institutional Animal Care and Use Committee. Twenty-four male 17 week old Sprague–Dawley rats with weights ranging from 369 to 408 g were utilized for our experiments. They were housed two per cage and maintained in a controlled environment. All animals had unrestricted access to food and water.

#### 2.11.1 Screw Implantation Surgery

The procedure for implant placement was similar to that used in the literature.^35,59,60^ Briefly, using anesthetized rats, an 10 mm incision was made on the craniomedial aspect of the proximal tibia, and blunt dissection was performed to expose the bone. A 1.1 mm diameter hole was drilled into the tibia using a 1.1 × 100 mm^2^ drill bit. One of three titanium implants was screwed into the right tibia. The rats were randomly placed into one of three groups, namely, uncoated bare metal screw, PLGA polymer only coated screw, or ZA/PLGA-coated screw with eight animals in each group. Once screws were implanted, 4–0 Vicryl absorbable sutures (Ethicon, Somerville, NJ) were subcutaneously placed to close the incision. Animals were monitored for the duration of the 8 week experiment.

#### 2.11.2 MicroCT Imaging

After 8 weeks, the animals were euthanized, and *in vivo* three-dimensional (3D) imaging was performed on all the animals using microCT (Bruker Biospin SkyScan 1176; Billerica, MA). In 3D, the spatial resolution was 9 μm. The system source was set to 65 kV, 385 μA, and 1037 ms exposure. A Dell Workstation R5500 mounted inside the scanner was used for acquisition control and 3D reconstruction (Windows 7 Professional, 64-bit). NRecon software was utilized for reconstruction (Modified Feldkamp multislice volumetric reconstruction algorithm for single slice or full cross section sized reconstructions) and postcapture image processing. DataViewer software was used for viewing images slice-by-slice. Two-dimensional MicroCT images were analyzed using ImageJ image processing software (version 1.8.0; NIH, Bethesda, MD). MicroCT images were analyzed in their original 8-bit (grayscale) format without any additional adjustment. The scale of the images was set based on the dimensions of the *in vivo* screw implants with the screw length being defined as 8.0 mm. Regions of interest (ROIs) were then manually selected for quantification using polygon selections. The ROIs were defined as the medullary regions from the screw crest to the 1 mm distance away from the screw crest; these ROIs were selected for both the proximal and the distal aspects of the screw crest. Mean gray value for each sample was based on the average of total ROI measurements made in the proximal and the distal aspects of the same sample. GraphPad Prism software (version 9.4.0; La Jolla, CA) was used to analyze the measured mean gray values for statistical differences using one-way analysis of variance (ANOVA), and subsequently Tukey’s posthoc test for comparison between implant coating types.

#### 2.11.3 Histology

First, the screws were removed from the tibiae of each animal. The bone tissue was prepared for sectioning by first fixing the tissue for at least 24 h in 10% neutral buffered formalin. Then the tissue was rinsed with distilled water multiple times for 30 min and underwent a decalcification step using 5% formic acid to avoid torn or ragged sections during the microtome process. The tissue was then left in the decalcification solution, and this was changed at least every other day until no residual calcification could be detected using the Faxitron OR Specimen Radiography System (Hologic, Marlborough, MA). Once decalcification was complete, the tissue was rinsed again with distilled water and stored in 70% alcohol. The decalcified and paraffin embedded tissues were sliced in the coronal plane using a microtome, then mounted and stained with Masson’s Trichome and hematoxylin and eosin (H&E) stains. The bright field images were used to assess new bone growth.

## 3 Results and Discussion

### 3.1 Characterization of ZA/Ca/DOPA, ZA/AF647-ZOL/Ca/DOPA, and AF-ZOL/Ca/DOPA Composites

The ZA drug contents of the ZA/Ca/DOPA and ZA/AF647-ZOL/Ca/DOPA composites were assessed using IE-HPLC. The concentration of ZA in the ZA/Ca/DOPA composites suspension used as a coating solution for the *in vivo* study was 682.7 μg/mL, while for the ZA/AF647-ZOL/Ca/DOPA composites suspension used as a coating solution for IVIS imaging, the concentration of ZA was 45.9 μg/mL. Since the ZA/Ca/DOPA composites used for the *in vivo* study were a combination of 11 batches of composites, the %EE was calculated for four representative batches of ZA/Ca/DOPA composites (Table 1). The quantified ZA concentrations in these four representative batches ranged from 272.7 to 379.3 μg/mL, and the %EE values for these representative batches ranged from 13.92 to 19.35%. TEM was used to characterize the morphology of the ZA/Ca/DOPA composites.

**Table 1:** Sample Concentrations and %EE Values for Four Representative Batches of ZA/Ca/DOPA Composites, the ZA/AF647-ZOL/Ca/DOPA Composites Used for IVIS Imaging, and the AF647-ZOL/Ca/DOPA Composites^a^

composite sample	ZA concentration of composite suspension (μg/mL)	%EE for ZA	AF647-ZOL concentration of composite suspension (ng/mL)	%EE for AF647-ZOL
ZA/Ca/DOPA Batch 1	379.3 ± 2.0	19.35 ± 0.10	N/A	N/A
ZA/Ca/DOPA Batch 2	272.7 ± 8.2	13.92 ± 0.42	N/A	N/A
ZA/Ca/DOPA Batch 3	332.0 ± 0.8	16.94 ± 0.04	N/A	N/A
ZA/Ca/DOPA Batch 4	299.5 ± 6.3	15.28 ± 0.32	N/A	N/A
ZA/AF647-ZOL/Ca/DOPA	45.9 ± 1.9	6.50 ± 0.003	12.75	1.5
AF647-ZOL/Ca/DOPA	N/A	N/A	32.28	0.5

a The ZA concentrations were determined using IE-HPLC and the AF647-ZOL concentration was determined using fluorescence spectroscopy. N/A indicates measurements that were not applicable for that particular formulation. Data presented as mean ± standard deviation (*n* = 2) for ZA concentration of composite suspension and %EE for ZA. For %EE and concentration of AF647-ZOL, *n* = 1.

**Figure 3:**
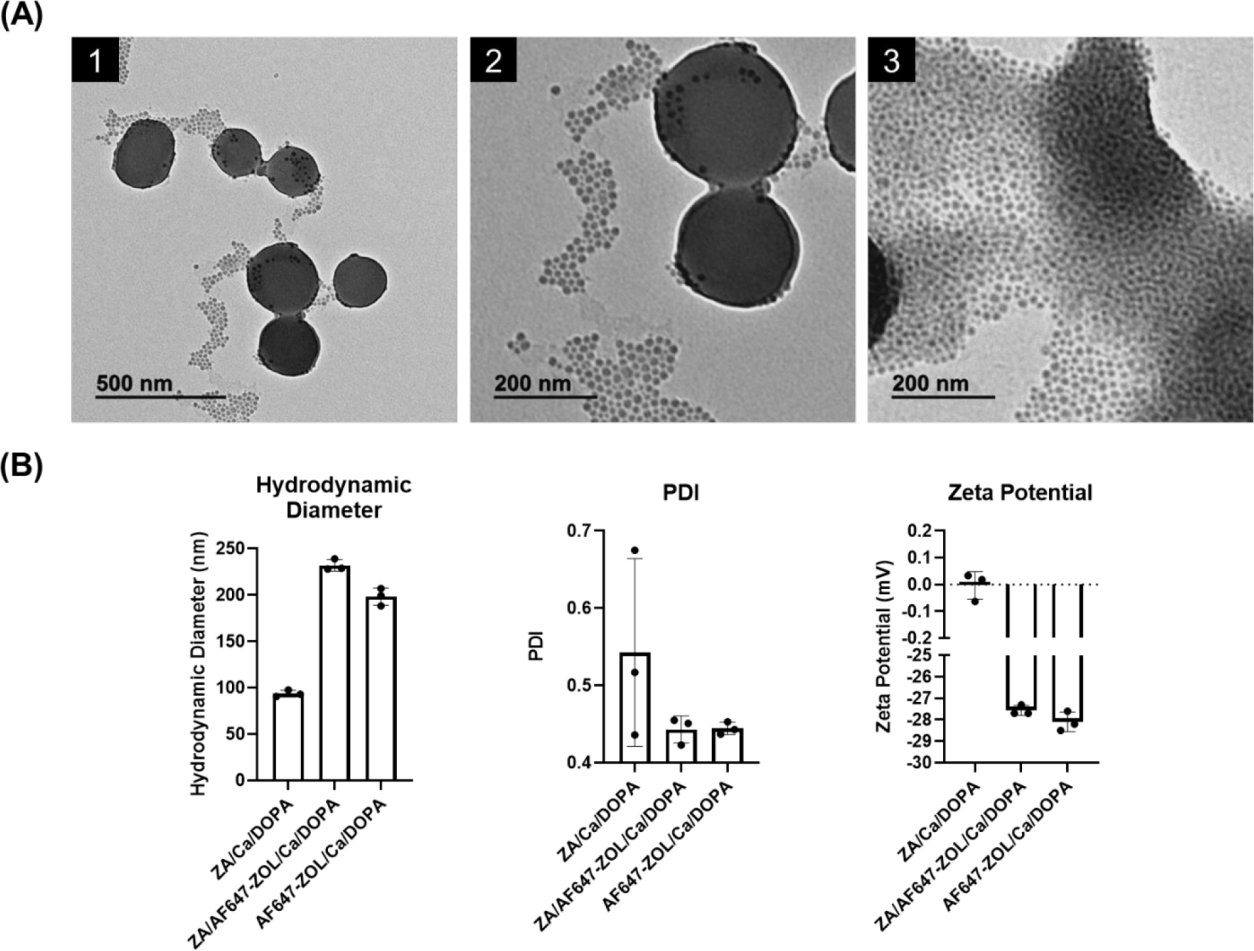
Characterization of ZA/Ca/DOPA, ZA/AF647-ZOL/Ca/DOPA, and AF647-ZOL/Ca/DOPA composites. (A) TEM micrographs of a single batch of ZA/Ca/DOPA composites coated with DSPE-PEG_2K_ with images taken at different magnifications. Scale bars represent 500 nm for A1 micrograph and 200 nm for A2 and A3 micrographs. (B) Graphs displaying the mean hydrodynamic diameter, PDI, and zeta potential for ZA/Ca/DOPA, ZA/AF647-ZOL/Ca/DOPA, and AF647-ZOL/Ca/DOPA composites, the latter two being used for IVIS imaging and *in vitro* release, respectively. Data are represented as mean ± SD with the individual data points superimposed (*n* = 3).

TEM micrographs of the ZA/Ca/DOPA composites showed that they were spherical, uniform and polydisperse (Figure 3A). Most of the composites had a size of ∼50 nm, with a few larger spherical composites of ∼200 nm in diameter. These images confirmed the size distribution indicated by the Zetasizer measurements for these composites.

The AF647-ZOL concentrations for the ZA/AF647-ZOL/Ca/DOPA composites used for IVIS imaging and the AF647-ZOL/Ca/DOPA composites used for the *in vitro* release study were characterized by comparing the fluorescence of the DSPE-PEG_2K_-coated samples to a standard curve of aqueous AF647-ZOL solutions with known concentrations ranging from 0.016 to 0.258 μg/mL, as well as a sample of Nanopure water alone as a blank (Figure S1). The AF647-ZOL concentrations were found to be 12.75 and 32.28 ng/mL for the ZA/AF647-ZOL/Ca/DOPA and AF647-ZOL/Ca/DOPA composites, respectively. The %EE for the ZA encapsulation within the ZA/AF647-ZOL/Ca/DOPA composites was found to be 6.5%. The %EE values for the AF647-ZOL encapsulation for the ZA/AF647-ZOL/Ca/DOPA and AF647-ZOL/Ca/DOPA composites were 1.5 and 0.5%, respectively (Table 1). The decreased encapsulation efficiency for the composites which included the AF647-ZOL compound can be attributed to the fact that the AF647-ZOL compound is significantly larger in size compared to ZA. This difference would hinder the ability to load the compound into RMs. The original RM protocol did not load AF647-ZOL into the composite, but instead, simply injected it in conjunction with the ZA/Ca/DOPA composites at a concentration of 2% (*w*/*w*) compared to the ZA added.^53^ The synthesis of these ZA/AF647-ZOL/Ca/DOPA and AF647-ZOL/Ca/DOPA is a novel protocol, so no previously published results are available in the literature.

The Zetasizer Nano ZS particle analyzer was used to determine the hydrodynamic diameter, PDI, and zeta potential for the ZA/Ca/DOPA composites, as well as the ZA/AF647-ZOL/Ca/DOPA and AF647-ZOL/Ca/DOPA composites (Figure 3B). The mean hydrodynamic diameter, PDI, and zeta potential for the ZA/Ca/DOPA composites were 93.7 ± 3.6 nm, 0.543 ± 0.122, and −0.004 ± 0.052 mV, respectively. The ZA/AF647-ZOL/Ca/DOPA composites had mean hydrodynamic diameter, PDI, and zeta potential values of 232.0 ± 6.2 nm, 0.443 ± 0.017, and −27.57 ± 0.23 mV, respectively. The AF647-ZOL/Ca/DOPA composites had mean hydrodynamic diameter, PDI, and zeta potential values of 198.1 ± 9.3 nm, 0.444 ± 0.008, and −28.10 ± 0.46 mV, respectively. The increased size of the ZA/AF647-ZOL/Ca/DOPA and AF647-ZOL/Ca/DOPA composites is likely due to the significantly increased size of the compound being loaded, compared to ZA. The negative charges of the composites were caused by the presence of several sulfonic acid (SO_3_H) functional groups branching out from the AlexaFluor647 probe. Likely they were strong enough to overcome the net neutral charge of the DSPE-PEG_2K_, thus the net neutral lipid was not as effective at neutralizing the overall charge of those composites compared to the ZA/Ca/DOPA composites.

### 3.2 Coating Composition and Technique

ZA/AF647-ZOL/Ca/DOPA, AF647-ZOL/Ca/DOPA, and ZA/Ca/DOPA composites were used to make coating solutions using the coating composition protocol previously described in section 2.8. Using IE-HPLC and fluorescence spectroscopy the concentrations of each compound in their respective coating solutions were calculated. The ZA/AF67-ZOL/PLGA coating solution had a ZA concentration of 15.3 μg/mL and an AF647-ZOL concentration of 4.25 ng/mL. These values equate to an AF647-ZOL/ZA (μg/μg) ratio of 0.00027. The AF67-ZOL/PLGA coating solution had an AF647-ZOL concentration of 10.76 ng/mL. The ZA/PLGA coating solution had a ZA concentration of 227.6 μg/mL. These three coating solutions were used to successfully coat model titanium screws for *in vitro* and *in vivo* studies.

### 3.3 IVIS Imaging ZA/AF647-ZOL/PLGA-Coated Screws

In order to confirm successful ZA drug deposition onto coated screws, IVIS imaging (Figure S2A) was used to determine the fluorescence intensity of an uncoated screw, coated screw, remaining coating solution after the dip-coating process, and several aqueous AF647-ZOL solutions with concentrations ranging from 10.55 ng/mL to 0.13 μg/mL, which were plotted into a standard curve (Figure S2B). The volumes of each of the standard solutions was kept constant at 200 μL; therefore, the amount of AF647-ZOL in each sample was easily calculated. The amount of AF647-ZOL that was coated onto the screw was deduced to be 0.48 ng. These results demonstrate that the drug, as well as the PLGA polymer, were successfully deposited onto the screw surface. By using the fluorescently labeled version of the compound, at least 0.48 ng of the AF647-ZOL was deposited onto the screw surface. Since the ratio of AF647-ZOL:ZA for the ZA/AF647-ZOL/Ca/DOPA composites was found to be 0.00027, this would mean that there was approximately 1.78 μg of ZA deposited onto the screw surface.

### 3.4 Release Study using AF647-ZOL/PLGA-Coated Screws

**Figure 4:**
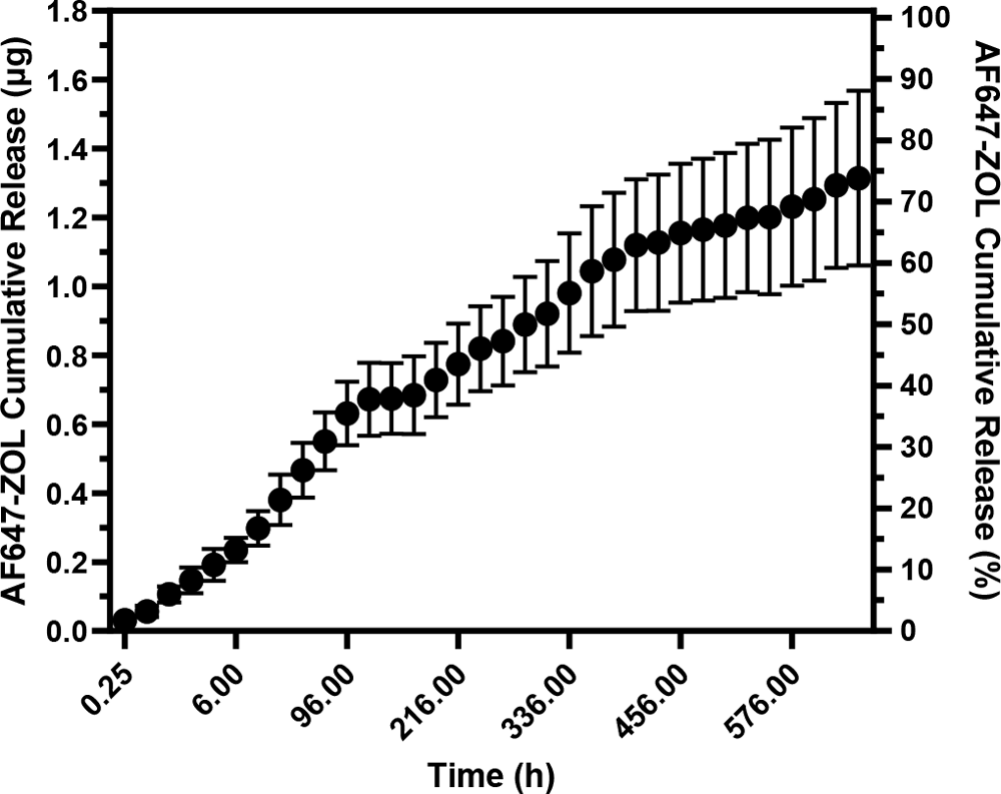
*In vitro* cumulative release study. Six AF647-ZOL/PLGA-coated screws were individually placed into a 24-well plate and left in 600 μL of PBS over one month. Measurements were made as described in section 2.10. Data are represented as mean ± SD (*n* = 6).

The release of AF647-ZOL from six AF647-ZOL/PLGA-coated titanium screws individually submerged in PBS was measured over the course of 1 month. The cumulative AF647-ZOL release profile obtained from the study results are shown in Figure 4. The release profile was biphasic, with an initial burst release of 0.47 μg occurring within 48 h, followed by a gradual sustained release of approximately 0.8 μg of AF647-ZOL over the remaining 528 h. The cumulative amount of AF647-ZOL that was released from the coated screws reached 1.31 μg by the end of the study, with 30% of that amount being release within the first 48 h. The burst release was likely due to ZA present at the surface of the screw coating. Ideally, zero order release kinetics are generally desired, and a preincubation step (e.g., in sterile saline), prior to implanting the screw may result in reducing the burst release and thus warrants future investigation.

### 3.5 Animal Study Results

#### 3.5.1 MicroCT Imaging

**Figure 5:**
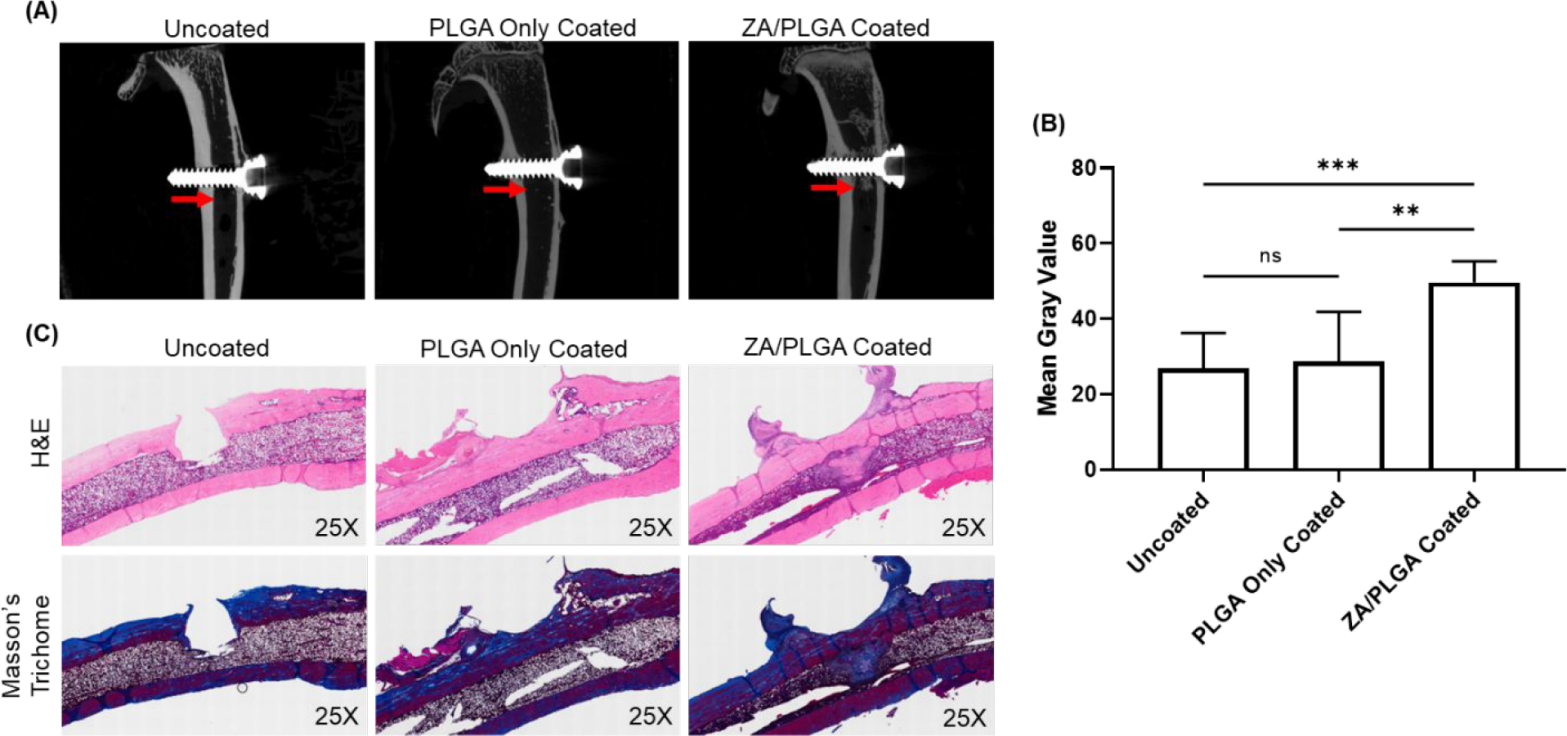
*In vivo* studies showing the results of implantation with titanium screws of various coatings. (A) MicroCT images of rat tibiae implanted with indicated titanium screws. The red arrow is pointing at the medullary cavity of the tibia and specifically the location where new bone growth was seen in the ZA/PLGA animals. (B) Mean gray values of the medullary regions from the screw crest to the 1 mm distance away from the screw crest; ROIs were selected for both the proximal and the distal aspects of the screw crest. *P* values were determined by one-way ANOVA with Tukey’s posthoc test; **, *P* < 0.005; ***, *P* < 0.0005. *N* = 8 rats per group. (C) Masson’s Trichome and H&E stained histological images of tibiae from rats implanted with indicated titanium screws. All histological images are displayed at 25× magnification.

MicroCT images of the animals were obtained after euthanasia at 8 weeks. Figure 5A shows representative microCT images for one animal per group. From the microCT results, it was seen that the animals implanted with ZA/PLGA-coated screws showed new bone growth in the medullary cavity of the rat tibiae. This effect was not seen for the uncoated or PLGA only coated groups. Figure 5B shows the quantification of the microCT images using ImageJ in terms of mean gray values in the medullary regions in the 1 mm vicinity of the screw crest. The measured mean gray values represent the extent of new bone growth in the regions with the animals implanted with ZA/PLGA-coated screws exhibiting significantly more bone growth in the medullary cavity than the animals implanted with the uncoated screws or the PLGA only coated screws. The fact that new bone was clearly evident in the ZA/PLGA animals is a positive indication that there was enough of the drug remaining at the site to promote bone growth around the implant site.

#### 3.5.2 Histology

Histological images were taken after staining tissues with Masson’s trichome and H&E stains (Figure 5C). The Masson’s trichome stain is used to distinguish osteoid seams and muscle (red) from mineralized bone (blue), and nuclei (dark gray). The H&E stains the nuclei blue/purple while the cytoplasm and different tissues and cell components are colored various shades of pink. Histological results for the rats given the uncoated screw showed bone loss at the site of the screw implant. There was significant loss of bone at the internal spongy bone of the medullary cavity, cortex, and external periosteum due to the implant. The animals implanted with the PLGA only coated screw showed normal spongy bone within the medullary cavity and increased mineralized bone growth at the cortex and periosteum within the implant site area, compared to the uncoated screw samples. The animals implanted with the ZA/PLGA-coated screw displayed new bone growth, which was distinct from the naturally occurring medullary cavity spongy bone but instead resembled the cortical bone. This mineralized bone growth spread throughout the length of the medullary cavity toward the periosteum along the region where the implant was present. The animals implanted with the ZA/PLGA-coated screws were the only animals that showed new bone growth around the implant site which confirmed the results seen from the microCT images.

## 4 Conclusions

In this work, ZA/Ca/DOPA, ZA/AF647-ZOL/Ca/DOPA, and AF647-ZOL/Ca/DOPA composites were successfully synthesized using the reverse emulsion method, yielding nanosized composite particles with mean hydrodynamic diameters ranging from ∼90–230 nm, depending on the type of zaledronate compound loaded. The composites were dispersed in a PLGA solution and subsequently coated onto titanium screws, conferring a desired sustained release profile of AF647-ZOL over 1 month *in vitro* with a minimal burst release of the coated AF647-ZOL over the first 48 h of the *in vitro* release study. An *in vivo* study showed that the ZA/PLGA-coated screws promoted new bone growth in the medullary cavity of the rats’ tibiae as opposed to the evident bone loss in the implant area in the case for uncoated screws. The promotion of local medullary bone growth by the ZA/PLGA-coated screws signifies local release of zoledronate and suggests a potential for their use as novel coated bone implants to improve osseointegration. The use of PLGA as a coating medium also allows for the possibility of incorporating other therapeutic agents into the coating to improve bone regeneration and osseointegration, and allows for the opportunity to optimize the release rate by adjusting the polymer’s lactic acid to glycolic acid ratio. Future studies and ongoing studies may involve investigating alternative coating methods, such as electrospray deposition, plasma spraying, electrophoretic deposition, or pulsed laser deposition, to further fine-tune the release of zoledronate from the coating layer. Additional biomechanical studies to measure the osteointegration of the coated screws, such as pull-out force and removal torque experiments, would provide valuable information to further characterize the benefits of this novel coating. As OA usually occurs in the load-bearing joints such as the knee and the hip, subsequent studies should consider the use of loaded models for the assessment of osseointegration to better represent the clinical settings of joint arthroplasty in OA.

## Supporting Information Available

AF647-ZOL standard curve for the release study; characterization of the ZA/AF647-ZOL/PLGA-coated screws using IVIS (PDF)

## References

[ref1] Pearle A. D.; Warren R. F.; Rodeo S. A. Basic science of articular cartilage and osteoarthritis. Clin Sports Med. 2005, 24 (1), 1–12. 10.1016/j.csm.2004.08.007. 15636773

[ref2] Zhang W.; Ouyang H.; Dass C. R.; Xu J. Current research on pharmacologic and regenerative therapies for osteoarthritis. Bone Res. 2016, 4, 15040 10.1038/boneres.2015.40. 26962464 PMC4772471

[ref3] Martel-Pelletier J.; Barr A. J.; Cicuttini F. M.; Conaghan P. G.; Cooper C.; Goldring M. B.; Goldring S. R.; Jones G.; Teichtahl A. J.; Pelletier J. P. Osteoarthritis. Nat. Rev. Dis Primers 2016, 2, 16072 10.1038/nrdp.2016.72. 27734845

[ref4] Murphy N. J.; Eyles J. P.; Hunter D. J. Hip Osteoarthritis: Etiopathogenesis and Implications for Management. Adv. Ther 2016, 33 (11), 1921–1946. 10.1007/s12325-016-0409-3. 27671326 PMC5083776

[ref5] Gademan M. G.; Hofstede S. N.; Vliet Vlieland T. P.; Nelissen R. G.; Marang-van de Mheen P. J. Indication criteria for total hip or knee arthroplasty in osteoarthritis: a state-of-the-science overview. BMC Musculoskelet Disord 2016, 17 (1), 463 10.1186/s12891-016-1325-z. 27829422 PMC5103467

[ref6] Kim D. M.; Han M.; Jeon I. H.; Shin M. J.; Koh K. H. Range-of-motion improvement and complication rate in open and arthroscopic osteocapsular arthroplasty for primary osteoarthritis of the elbow: a systematic review. Int. Orthop 2020, 44 (2), 329–339. 10.1007/s00264-019-04458-z. 31834444

[ref7] Delco M. L.; Kennedy J. G.; Bonassar L. J.; Fortier L. A. Post-traumatic osteoarthritis of the ankle: A distinct clinical entity requiring new research approaches. J. Orthop Res. 2017, 35 (3), 440–453. 10.1002/jor.23462. 27764893 PMC5467729

[ref8] Agarwal R.; Garcia A. J. Biomaterial strategies for engineering implants for enhanced osseointegration and bone repair. Adv. Drug Deliv Rev. 2015, 94, 53–62. 10.1016/j.addr.2015.03.013. 25861724 PMC4598264

[ref9] Goodman S. B.; Yao Z.; Keeney M.; Yang F. The future of biologic coatings for orthopaedic implants. Biomaterials 2013, 34 (13), 3174–83. 10.1016/j.biomaterials.2013.01.074. 23391496 PMC3582840

[ref10] Campbell A. A.; Song L.; Li X. S.; Nelson B. J.; Bottoni C.; Brooks D. E.; DeJong E. S. Development, characterization, and anti-microbial efficacy of hydroxyapatite-chlorhexidine coatings produced by surface-induced mineralization. J. Biomed Mater. Res. 2000, 53 (4), 400–7. 10.1002/1097-4636(2000)53:4<400::AID-JBM14>3.0.CO;2-Z. 10898881

[ref11] Hamoudi-Ben Yelles M. C.; Tran Tan V.; Danede F.; Willart J. F.; Siepmann J. PLGA implants: How Poloxamer/PEO addition slows down or accelerates polymer degradation and drug release. J. Controlled Release 2017, 253, 19–29. 10.1016/j.jconrel.2017.03.009. 28284831

[ref12] Ray S.; Acharya R.; Saha S.; Islam A.; Dey S.; Nandi S. K.; Mandal T. K.; Banerjee G.; Chakraborty J. Role of a nitrogenous bisphosphonate (local delivery) incorporated vitreous coating (with/without polymer) on surgical grade SS316L implant material to improve fixation at the damaged tissue site. Rsc Adv. 2016, 6 (92), 89467–89483. 10.1039/C6RA13155G.

[ref13] Ding W. Opportunities and challenges for the biodegradable magnesium alloys as next-generation biomaterials. Regen Biomater 2016, 3 (2), 79–86. 10.1093/rb/rbw003. 27047673 PMC4817317

[ref14] Ballarre J.; Desimone P. M.; Chorro M.; Baca M.; Orellano J. C.; Cere S. M. Bone quality around bioactive silica-based coated stainless steel implants: analysis by micro-Raman, XRF and XAS techniques. J. Struct Biol. 2013, 184 (2), 164–72. 10.1016/j.jsb.2013.09.016. 24076155

[ref15] Guillot R.; Pignot-Paintrand I.; Lavaud J.; Decambron A.; Bourgeois E.; Josserand V.; Logeart-Avramoglou D.; Viguier E.; Picart C. Assessment of a polyelectrolyte multilayer film coating loaded with BMP-2 on titanium and PEEK implants in the rabbit femoral condyle. Acta Biomater 2016, 36, 310–22. 10.1016/j.actbio.2016.03.010. 26965394 PMC5015710

[ref16] Kim S. M.; Kang M. H.; Kim H. E.; Lim H. K.; Byun S. H.; Lee J. H.; Lee S. M. Innovative micro-textured hydroxyapatite and poly(l-lactic)-acid polymer composite film as a flexible, corrosion resistant, biocompatible, and bioactive coating for Mg implants. Mater. Sci. Eng. C Mater. Biol. Appl. 2017, 81, 97–103. 10.1016/j.msec.2017.07.026. 28888023

[ref17] Actis L.; Gaviria L.; Guda T.; Ong J. L. Antimicrobial surfaces for craniofacial implants: state of the art. J. Korean Assoc Oral Maxillofac Surg 2013, 39 (2), 43–54. 10.5125/jkaoms.2013.39.2.43. 24471018 PMC3858148

[ref18] Apostu D.; Lucaciu O.; Lucaciu G. D.; Crisan B.; Crisan L.; Baciut M.; Onisor F.; Baciut G.; Campian R. S.; Bran S. Systemic drugs that influence titanium implant osseointegration. Drug Metab Rev. 2017, 49 (1), 92–104. 10.1080/03602532.2016.1277737. 28030966

[ref19] Evans J. T.; Evans J. P.; Walker R. W.; Blom A. W.; Whitehouse M. R.; Sayers A. How long does a hip replacement last? A systematic review and meta-analysis of case series and national registry reports with more than 15 years of follow-up. Lancet 2019, 393 (10172), 647–654. 10.1016/S0140-6736(18)31665-9. 30782340 PMC6376618

[ref20] Evans J. T.; Walker R. W.; Evans J. P.; Blom A. W.; Sayers A.; Whitehouse M. R. How long does a knee replacement last? A systematic review and meta-analysis of case series and national registry reports with more than 15 years of follow-up. Lancet 2019, 393 (10172), 655–663. 10.1016/S0140-6736(18)32531-5. 30782341 PMC6381229

[ref21] Koob S.; Gaertner F. C.; Jansen T. R.; Schmolders J.; Gravius S.; Strunk H.; Wirtz D. C.; Essler M. Diagnosis of peri-prosthetic loosening of total hip and knee arthroplasty using ^18^F-Fluoride PET/CT. Oncotarget 2019, 10 (22), 2203–2211. 10.18632/oncotarget.26762. 31040911 PMC6481340

[ref22] Schwartz A. M.; Farley K. X.; Guild G. N.; Bradbury T. L. Jr. Projections and Epidemiology of Revision Hip and Knee Arthroplasty in the United States to 2030. J. Arthroplasty 2020, 35 (6S), S79–S85. 10.1016/j.arth.2020.02.030. 32151524 PMC7239745

[ref23] Jakobsen T.; Kold S.; Shiguetomi-Medina J.; Baas J.; Soballe K.; Rahbek O. Topical zoledronic acid decreases micromotion induced bone resorption in a sheep arthroplasty model. BMC Musculoskelet Disord 2017, 18 (1), 441 10.1186/s12891-017-1802-z. 29132335 PMC5683542

[ref24] Albrektsson T.; Chrcanovic B.; Jacobsson M.; Wennerberg A. Osseointegration of Implants: A Biological and Clinical Overview. JSM Dent. Surg. 2017, 2 (3), 1–6.

[ref25] Mavrogenis A. F.; Dimitriou R.; Parvizi J.; Babis G. C. Biology of implant osseointegration. J. Musculoskeletal Neuronal Interact. 2009, 9 (2), 61–71. 19516081

[ref26] Shi L.; Shen K.; Chu L.; Yu K. X.; Yu Q. S.; Deng R.; Deng Z. L. Biomechanical Study of Novel Unilateral Fixation Combining Unilateral Pedicle and Contralateral Translaminar Screws in the Subaxial Cervical Spine. World Neurosurg. 2019, 121, e684–e690. 10.1016/j.wneu.2018.09.191. 30292663

[ref27] Ong K. L.; Yun B. M.; White J. B. New biomaterials for orthopedic implants. Orthopedic Research and Reviews 2015, 7, 107–130. 10.2147/ORR.S63437.

[ref28] Smeets R.; Stadlinger B.; Schwarz F.; Beck-Broichsitter B.; Jung O.; Precht C.; Kloss F.; Grobe A.; Heiland M.; Ebker T. Impact of Dental Implant Surface Modifications on Osseointegration. Biomed Res. Int. 2016, 6285620 10.1155/2016/6285620. 27478833 PMC4958483

[ref29] Ogle O. E. Implant surface material, design, and osseointegration. Dent Clin North Am. 2015, 59 (2), 505–20. 10.1016/j.cden.2014.12.003. 25835806

[ref30] Ray S.; Acharya R.; Saha S.; Islam A.; Dey S.; Nandi S. K.; Mandal T. K.; Banerjee G.; Chakraborty J. Role of a nitrogenous bisphosphonate (local delivery) incorporated vitreous coating (with/without polymer) on surgical grade SS316L implant material to improve fixation at the damaged tissue site. Rsc Adv. 2016, 6 (92), 89467–89483. 10.1039/C6RA13155G.

[ref31] Gong L.; Altman R. B.; Klein T. E. Bisphosphonates pathway. Pharmacogenet Genomics 2011, 21 (1), 50–3. 10.1097/FPC.0b013e328335729c. 20023594 PMC3086066

[ref32] Raichur V.; Vemula K. D.; Bhadri N.; Razdan R. Zolendronic Acid-Conjugated PLGA Ultrasmall Nanoparticle Loaded with Methotrexate as a Supercarrier for Bone-Targeted Drug Delivery. AAPS PharmSciTech 2017, 18 (6), 2227–2239. 10.1208/s12249-016-0691-z. 28070850

[ref33] Qi M.; Hu J.; Li J.; Li J.; Dong W.; Feng X.; Yu J. Effect of zoledronate acid treatment on osseointegration and fixation of implants in autologous iliac bone grafts in ovariectomized rabbits. Bone 2012, 50 (1), 119–27. 10.1016/j.bone.2011.10.011. 22023930

[ref34] Skerjanec A.; Berenson J.; Hsu C.; Major P.; Miller W. H. Jr.; Ravera C.; Schran H.; Seaman J.; Waldmeier F. The pharmacokinetics and pharmacodynamics of zoledronic acid in cancer patients with varying degrees of renal function. J. Clin Pharmacol 2003, 43 (2), 154–62. 10.1177/0091270002239824. 12616668

[ref35] Chen B.; Li Y.; Yang X.; Xu H.; Xie D. Zoledronic acid enhances bone-implant osseointegration more than alendronate and strontium ranelate in ovariectomized rats. Osteoporos Int. 2013, 24 (7), 2115–21. 10.1007/s00198-013-2288-7. 23389695

[ref36] Li X.; Sun W.; Li J.; Wang M.; Zhang H.; Pei L.; Boyce B. F.; Wang Z.; Xing L. Clomipramine causes osteoporosis by promoting osteoclastogenesis via E3 ligase Itch, which is prevented by Zoledronic acid. Sci. Rep 2017, 7, 41358 10.1038/srep41358. 28145497 PMC5286409

[ref37] Peter B.; Pioletti D. P.; Laib S.; Bujoli B.; Pilet P.; Janvier P.; Guicheux J.; Zambelli P. Y.; Bouler J. M.; Gauthier O. Calcium phosphate drug delivery system: influence of local zoledronate release on bone implant osteointegration. Bone 2005, 36 (1), 52–60. 10.1016/j.bone.2004.10.004. 15664002

[ref38] Agholme F.; Andersson T.; Tengvall P.; Aspenberg P. Local bisphosphonate release versus hydroxyapatite coating for stainless steel screw fixation in rat tibiae. J. Mater. Sci. Mater. Med. 2012, 23 (3), 743–52. 10.1007/s10856-011-4539-5. 22203517

[ref39] Andersson T.; Agholme F.; Aspenberg P.; Tengvall P. Surface immobilized zoledronate improves screw fixation in rat bone: a new method for the coating of metal implants. J. Mater. Sci. Mater. Med. 2010, 21 (11), 3029–37. 10.1007/s10856-010-4154-x. 20857321

[ref40] Tengvall P.; Skoglund B.; Askendal A.; Aspenberg P. Surface immobilized bisphosphonate improves stainless-steel screw fixation in rats. Biomaterials 2004, 25 (11), 2133–8. 10.1016/j.biomaterials.2003.08.049. 14741628

[ref41] Back D. A.; Pauly S.; Rommel L.; Haas N. P.; Schmidmaier G.; Wildemann B.; Greiner S. H. Effect of local zoledronate on implant osseointegration in a rat model. BMC Musculoskelet Disord 2012, 13, 42 10.1186/1471-2474-13-42. 22439827 PMC3323428

[ref42] Jakobsen T.; Bechtold J. E.; Soballe K.; Jensen T.; Greiner S.; Vestermark M. T.; Baas J. Local delivery of zoledronate from a poly (D,L-lactide)-Coating increases fixation of press-fit implants. J. Orthop Res. 2016, 34 (1), 65–71. 10.1002/jor.22979. 26177742 PMC6326075

[ref43] Jakobsen T.; Bechtold J. E.; Soballe K.; Jensen T.; Vestermark M. T.; Baas J. Local delivery of zoledronate from a poly (d,l-lactide)-coating increases fixation of hydroxy-coated implants. J. Orthop Res. 2017, 35 (5), 974–979. 10.1002/jor.23219. 26925986 PMC6338069

[ref44] Yu N. Y.; Schindeler A.; Peacock L.; Mikulec K.; Baldock P. A.; Ruys A. J.; Little D. G. In vivo local co-delivery of recombinant human bone morphogenetic protein-7 and pamidronate via poly-D, L-lactic acid. Eur. Cell Mater. 2010, 20, 431–41. 10.22203/eCM.v020a35. 21181649

[ref45] Moon S. H.; Lee S. J.; Park I. S.; Lee M. H.; Soh Y. J.; Bae T. S.; Kim H. S. Bioactivity of Ti-6Al-4V alloy implants treated with ibandronate after the formation of the nanotube TiO2 layer. J. Biomed. Mater. Res. 2012, 100B (8), 2053–2059. 10.1002/jbm.b.32769. 22915455

[ref46] Ciolino J. B.; Hoare T. R.; Iwata N. G.; Behlau I.; Dohlman C. H.; Langer R.; Kohane D. S. A drug-eluting contact lens. Invest Ophthalmol Vis Sci. 2009, 50 (7), 3346–52. 10.1167/iovs.08-2826. 19136709 PMC4657544

[ref47] Fredenberg S.; Wahlgren M.; Reslow M.; Axelsson A. The mechanisms of drug release in poly(lactic-co-glycolic acid)-based drug delivery systems–a review. Int. J. Pharm. 2011, 415 (1–2), 34–52. 10.1016/j.ijpharm.2011.05.049. 21640806

[ref48] Jia H.; Kerr L. L. Sustained ibuprofen release using composite poly(lactic-co-glycolic acid)/titanium dioxide nanotubes from Ti implant surface. J. Pharm. Sci. 2013, 102 (7), 2341–8. 10.1002/jps.23580. 23657983

[ref49] Mylonaki I.; Strano F.; Deglise S.; Allemann E.; Alonso F.; Corpataux J. M.; Dubuis C.; Haefliger J. A.; Jordan O.; Saucy F.; Delie F. Perivascular sustained release of atorvastatin from a hydrogel-microparticle delivery system decreases intimal hyperplasia. J. Controlled Release 2016, 232, 93–102. 10.1016/j.jconrel.2016.04.023. 27091698

[ref50] Wilson G. J.; Marks A.; Berg K. J.; Eppihimer M.; Sushkova N.; Hawley S. P.; Robertson K. A.; Knapp D.; Pennington D. E.; Chen Y. L.; et al. The SYNERGY biodegradable polymer everolimus eluting coronary stent: Porcine vascular compatibility and polymer safety study. Cathet. Cardiovasc. Intervent. 2015, 86 (6), e247–e257. 10.1002/ccd.25993. 26009986

[ref51] Alexis F. Factors affecting the degradation and drug-release mechanism of poly(lactic acid) and poly[(lactic acid)-co-(glycolic acid)]. Polym. Int. 2005, 54 (1), 36–46. 10.1002/pi.1697.

[ref52] Li X.; Naguib Y. W.; Cui Z. In vivo distribution of zoledronic acid in a bisphosphonate-metal complex-based nanoparticle formulation synthesized by a reverse microemulsion method. Int. J. Pharm. 2017, 526 (1–2), 69–76. 10.1016/j.ijpharm.2017.04.053. 28455136 PMC5541900

[ref53] Li X.; Naguib Y. W.; Valdes S.; Hufnagel S.; Cui Z. Reverse Microemulsion-Based Synthesis of (Bis)phosphonate-Metal Materials with Controllable Physical Properties: An Example Using Zoledronic Acid-Calcium Complexes. ACS Appl. Mater. Interfaces 2017, 9 (16), 14478–14489. 10.1021/acsami.6b15006. 28252282 PMC5485920

[ref54] Zang X.; Zhang X.; Hu H.; Qiao M.; Zhao X.; Deng Y.; Chen D. Targeted Delivery of Zoledronate to Tumor-Associated Macrophages for Cancer Immunotherapy. Mol. Pharmaceutics 2019, 16 (5), 2249–2258. 10.1021/acs.molpharmaceut.9b00261. 30969779

[ref55] Au K. M.; Satterlee A.; Min Y.; Tian X.; Kim Y. S.; Caster J. M.; Zhang L.; Zhang T.; Huang L.; Wang A. Z. Folate-targeted pH-responsive calcium zoledronate nanoscale metal-organic frameworks: Turning a bone antiresorptive agent into an anticancer therapeutic. Biomaterials 2016, 82, 178–93. 10.1016/j.biomaterials.2015.12.018. 26763733 PMC4728024

[ref56] Junankar S.; Shay G.; Jurczyluk J.; Ali N.; Down J.; Pocock N.; Parker A.; Nguyen A.; Sun S.; Kashemirov B.; McKenna C. E.; Croucher P. I.; Swarbrick A.; Weilbaecher K.; Phan T. G.; Rogers M. J. Real-time intravital imaging establishes tumor-associated macrophages as the extraskeletal target of bisphosphonate action in cancer. Cancer Discov 2015, 5 (1), 35–42. 10.1158/2159-8290.CD-14-0621. 25312016 PMC4293349

[ref57] Li X.; Valdes S. A.; Alzhrani R. F.; Hufnagel S.; Hursting S. D.; Cui Z. Zoledronic Acid-containing Nanoparticles With Minimum Premature Release Show Enhanced Activity Against Extraskeletal Tumor. ACS Appl. Mater. Interfaces 2019, 11 (7), 7311–7319. 10.1021/acsami.8b16588. 30689348 PMC6582365

[ref58] Sun S.; Blazewska K. M.; Kadina A. P.; Kashemirov B. A.; Duan X.; Triffitt J. T.; Dunford J. E.; Russell R. G.; Ebetino F. H.; Roelofs A. J.; Coxon F. P.; Lundy M. W.; McKenna C. E. Fluorescent Bisphosphonate and Carboxyphosphonate Probes: A Versatile Imaging Toolkit for Applications in Bone Biology and Biomedicine. Bioconjug Chem. 2016, 27 (2), 329–40. 10.1021/acs.bioconjchem.5b00369. 26646666 PMC5117111

[ref59] Li Y.; Feng G.; Gao Y.; Luo E.; Liu X.; Hu J. Strontium ranelate treatment enhances hydroxyapatite-coated titanium screws fixation in osteoporotic rats. J. Orthop Res. 2010, 28 (5), 578–82. 10.1002/jor.21050. 20014319

[ref60] Li Y.; Gao Y.; Song G.; Liu X.; Hu J. Additive effects of estrogen replacement therapy and bisphosphonates on osseointegration of hydroxyapatite-coated titanium screws in ovariectomized rats. Oral Surg Oral Med. Oral Pathol Oral Radiol Endod 2010, 109 (5), 700–5. 10.1016/j.tripleo.2009.10.041. 20163971

