# Supplementary material for: Zoledronic Acid Implant Coating Results in Local Medullary Bone Growth
Source: Mol Pharm. 2022 Nov 15;19(12):4654–64. doi: 10.1021/acs.molpharmaceut.2c00644 (PMC9727731; doi:10.1021/acs.molpharmaceut.2c00644)
Supplement: Supplementary file 1 — mp2c00644_si_001.pdf [file mp2c00644_si_001.pdf]

Supporting Information

Novel Zoledronic Acid Implant Coating Results in Local Medullary Bone Growth

Juliana C. Quarterman[1], Pornpoj Phruttiwanichakun[1], Douglas C. Fredericks[2], Aliasger K. Salem[1,*]

[1] Department of Pharmaceutical Sciences and Experimental Therapeutics, College of Pharmacy, University of Iowa, Iowa City, IA 52242, USA

[2] The Bone Healing Research Laboratory, Department of Orthopedics and Rehabilitation, Carver College of Medicine, the University of Iowa, Iowa City, IA 52242, USA

*Corresponding author.
Email address: aliasger-salem@uiowa.edu (A. K. Salem)

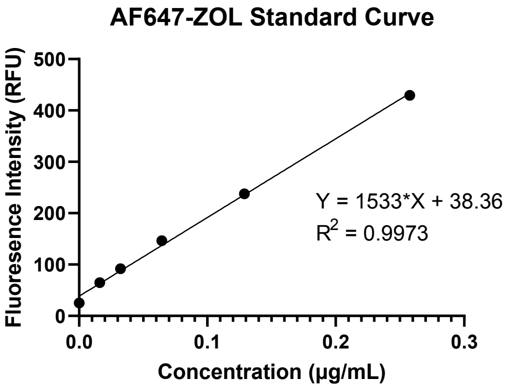

Supplementary Fig. S1 AF647-ZOL standard curve constructed using known concentrations of AF647-ZOL diluted in Nanopure water ranging from 0.016-0.26 μg/mL (5 concentrations). The units for fluorescence are relative fluorescence units (RFU).

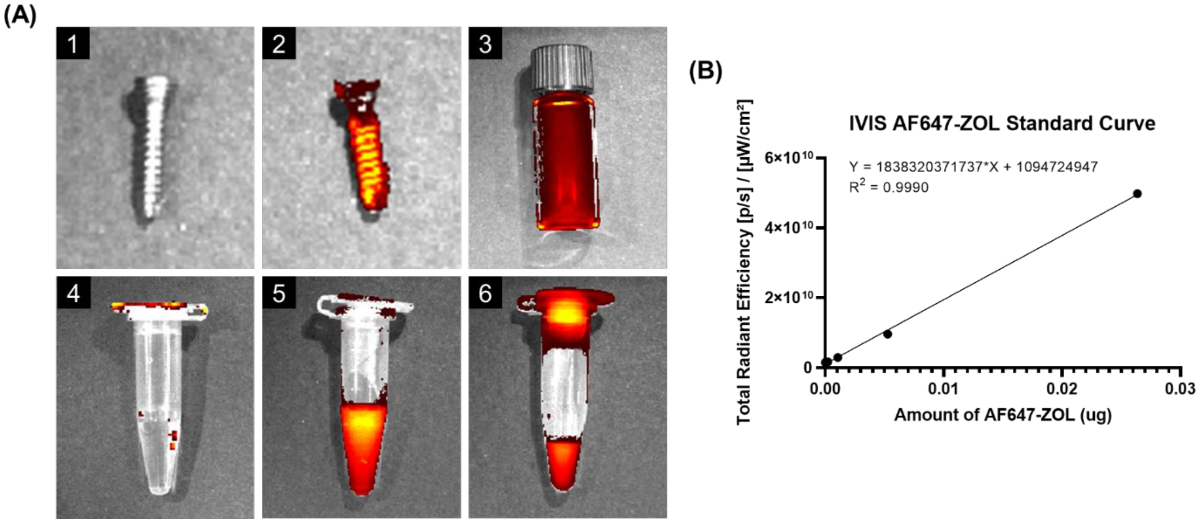

Supplementary Fig. S2 Characterization of the ZA/AF647-ZOL/PLGA coated screws. **(A)** Representative IVIS images of an uncoated screw (A1), coated screw (A2), remaining coating solution (A3), the highest (A5) and lowest (A6) concentrations of aqueous solutions of AF647-ZOL that were used to create the standard curve, and a sample of Nanopure water that was used as a blank (A4). The darker red color indicates higher intensity fluorescence compared to yellow (lower intensity). **(B)** Fluorescence intensity standard curve constructed from the IVIS measured fluorescence intensity found from standard solutions of AF647-ZOL in water with concentrations ranging from 0.211 ng/mL to 0.132 μg/mL (5 concentrations), which are equivalent to the range of 0.0422 ng- to 0.0264 μg-AF647-ZOL. The units for fluorescence on the IVIS instrument were [p/s] / [μW/cm²].
